# Primary squamous cell carcinoma of the kidney in southern Senegal (Ziguinchor): case report and review of the literature

**DOI:** 10.11604/pamj.2021.40.175.29222

**Published:** 2021-11-21

**Authors:** Fabrice Senghor, Ibou Thiam, Omar Sow, Aboubacar Traore, Boubacar Fall, Chérif Mohamed Moustapha Dial

**Affiliations:** 1 Pathological Anatomy Department, Aristide Le Dantec University Hospital, Dakar, Senegal,; 2 Health Sciences Training and Research Unit, Assane Seck University, Ziguinchor, Senegal,; 3 General Surgery Department, De La Paix University Hospital, Ziguinchor, Senegal,; 4 Urology Department, De La Paix University Hospital, Ziguinchor, Senegal,; 5 Pathological Anatomy Department, Idrissa Pouye General Hospital, Dakar, Senegal

**Keywords:** Kidney neoplasm, squamous cell carcinoma, metaplasia, Senegal, case report

## Abstract

The kidney's primary squamous cell carcinoma is a rare tumor, representing 0.5-0.8% of malignant renal tumors and 4% of upper urinary tract tumors. This pathology often occurs after a long past history of renal lithiasis and repeated untreated or poorly treated urinary tract infections. The delay in diagnosis resulting from an insidious symptomatology, without specific signs, often leads to a pejorative development, especially in poor countries. A seventy-nine-year-old Senegalese woman, with no past history of lithiasis nor recurrent urinary tract infection and urinary schistosomiasis, was received for a recurrent total hematuria associated with left lumbar pain. Clinical examination revealed a mobile tender left lumbar mass, with lumbar contact and renal sloshing. The left renal tumor´s diagnosis was retained on clinical and scannographic arguments, justifying an enlarged left total nephrectomy, by laparotomy. The anatomopathological examination of the surgical sample made it possible to make the diagnosis of primary invasive squamous cell carcinoma of the left kidney and to find foci of carcinoma in-situ on squamous metaplasia in the calyxes. Unlike the typical case of primary squamous cell carcinoma of the kidney, our patient did not have a long past history of renal lithiasis nor untreated or poorly treated recurrent urinary tract infections and urinary schistosomiasis. Primary squamous cell carcinoma of the kidney may not be related to a past history of recurrent urinary tract infections and lithiasis, but to any other cause of squamous metaplasia of the urothelium. Surgery remains the best option for this entity.

## Introduction

Primitive squamous cell carcinoma of the kidney is a rare malignant tumor often associated with squamous metaplasia due to chronic irritation. It represents 0.5 to 8% of malignant renal tumors [1] and 4% of tumors of the upper urinary tract [2]. This pathology often follows a long history of renal lithiasis and repeated untreated or poorly treated urinary tract infections [3,4]. The delay in diagnosis following an insidious symptomatology, without specific signs, often leads to a pejorative evolution. It is frequently discovered at advanced stages and is associated with a poor prognosis [5]. We report a case of renal squamous cell carcinoma followed in the peripheral zone in southern Senegal (Ziguinchor) with no history of lithiasis, recurrent urinary tract infection and urinary schistosomiasis, and insist on the epidemiological and anatomopathological characteristics of this histological type.

## Patient and observation

**Patient information**: it's about a 79-year-old woman with no particular medico-surgical history, in particular without a history of lithiasis or recurrent urinary tract infections and urinary schistosomiasis, who consulted for a recurrent total hematuria followed by left lumbar pain without irradiation, occurring intermittently and calmed by usual analgesics.

**Clinical findings**: the clinical examination showed a general condition distortion (ASA II “American Society of Anesthesiologists”) with presence of anorexia, a mobile and tender left lumbar mass, with lumbar contact and renal sloshing.

**Figure 1:**
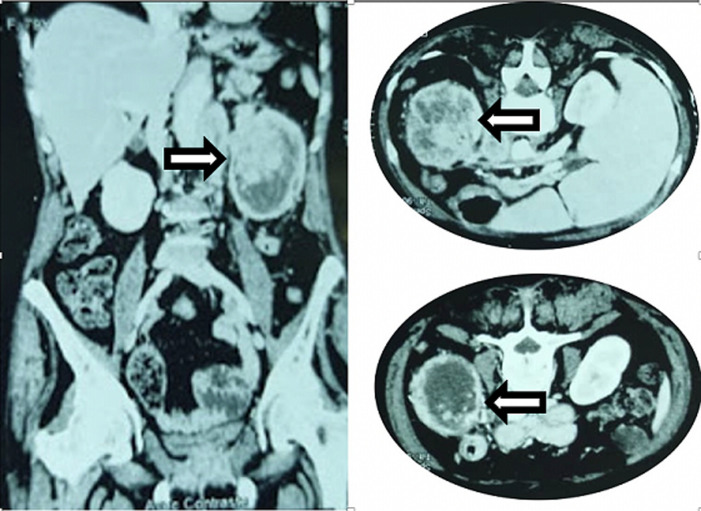
uroscanner - left kidney, site of a vascularized tissue mass of 8 cm long, spontaneously hypodense and strongly enhanced after a contrast product injection, with central necrosis and washing out late (arrows)

**Diagnostic assessment**: the patient had microcytic hypochromic anemia (7.9 g / dl) and impaired renal function (serum creatinine 18 mg/l; GFR “Glomerular filtration rate” 26.8 ml/min): Cyto-bacteriological examination of the urine was negative. The abdominal CT scan showed a left kidney, site of a vascularized tissue mass of 8 cm long, spontaneously hypodense and strongly enhanced after a contrast product injection, with central necrosis and washing out late. This formation was associated with a discrete superior calicar dilation and with left necrotic latero-aortic adenopathies. There was an absence of renal excretion on the left. The right kidney and other organs were normal (Figure 1). The renal tumor diagnosis was made according to clinical features and the scanning's results. The tumor was clinically classified as T2a, N1, Mx.

**Figure 2:**
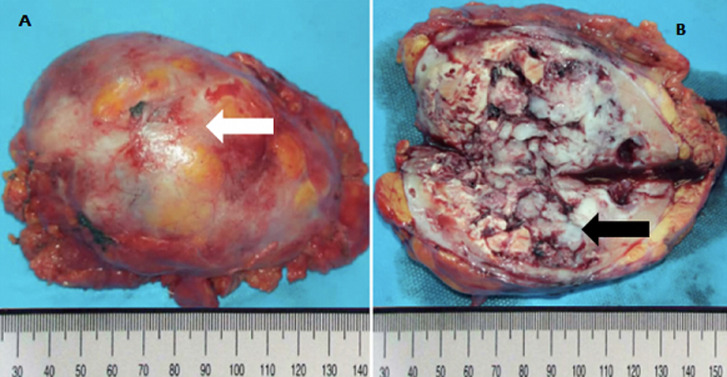
tumor left kidney (source: pathological cytology laboratory collection from “La Paix” University Hospital); A) fresh piece of left nephrectomy, showing an intact capsule (white arrow); B) section and opening of the left nephrectomy piece by its lateral edge showing a large tumor formation, yellowish white, associated with necrotic and hemorrhagic changes, occupying almost the entire kidney (black arrow)

**Figure 3:**
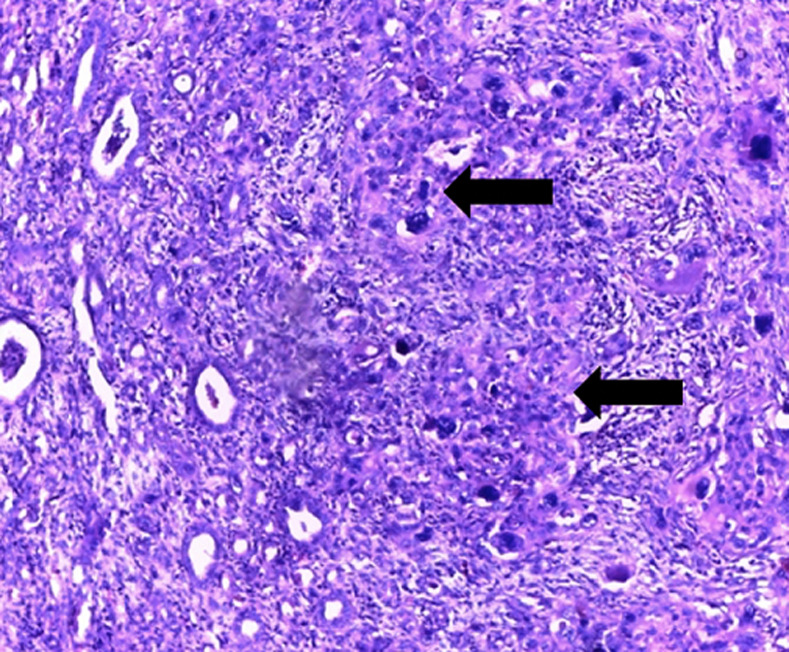
poorly differentiated squamous cell carcinoma with anaplastic focus of the left kidney; HE x100 (source: pathological cytology laboratory collection of the DANTEC CHU); often cohesive layers and clumps of atypical, poorly differentiated, dyskaryotic, hyperchromatic squamous epithelial cells, infiltrating a fibro-inflammatory stroma (arrows)

**Figure 4:**
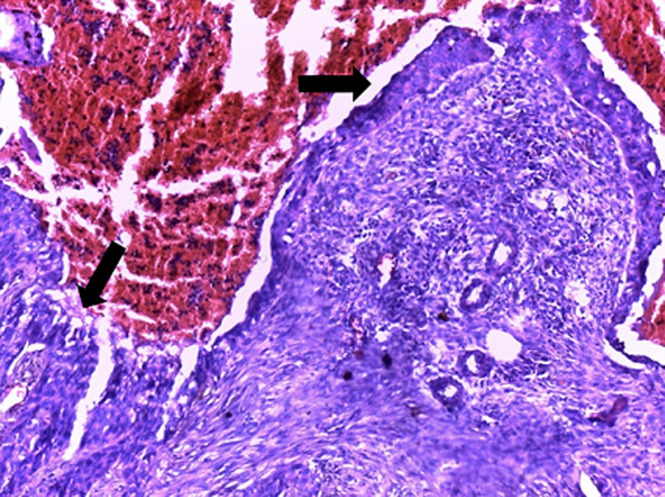
carcinoma in-situ vs high-grade dysplasia of the epithelial lining of the calyx; HE x 100 (source: pathological cytology laboratory collection from CHU le DANTEC) Malpighian metaplasia of the calyx urothelium, presenting with high-grade dysplasia versus carcinoma in-situ (arrows)

**Therapeutic intervention**: the patient underwent an enlarged radical nephrectomy which was done via midline laparotomy incision approach and TOLD´s fascia detachment allowing access to the kidney. Macroscopic examination objectified a left kidney weighing 200 grams and measuring 11.5 cm long axis. It was the site of a solid, fleshy, yellowish-white tumor formation of 11cm long, encompassing almost the entire kidney. It presented foci of central necrotic changes (Figure 2). The histology showed an infiltrating poorly differentiated squamous cell carcinoma (Figure 3), originating from the metaplastic epithelial lining of the calyxes (Figure 4). This proliferation infiltrated the hilum and renal tissue, in the form of clumps and layers of irregular polygonal epithelial cells contiguous. The nuclei were large, atypical, hyperchromatic and strongly nucleolated. Many figures of mitosis were observed. Anaplastic foci were noted in places. We noted the presence of epidermoidal metaplasia with high-grade dysplasia versus squamous cell carcinoma in situ at the level of the epithelial lining of the pelvis (Figure 4). The tumor extension was mainly local with damage to the capsule without breaking it, and no lymph node invasion was found (pT4N0Mx stage). The immediate postoperative consequences were simple.

**Follow-up and outcomes**: after 12 months´ follow-up, the abdominal CT scan was normal and renal function was maintained. To date, there is an absence of tumor recurrence.

## Discussion

Several other risk factors, different from lithiasis and recurrent upper urinary tract infections, are implicated in primitive squamous cell carcinomas of the kidney: notably tobacco, and urinary schistosomiasis [3,4,6,7]. In our case, the interrogation and the pathological examination could not highlight an associated risk factor. The average age of this cancer onset is between 56 and 69 years, without predominance of gender. Our patient was elderly (79 years old). Most common symptoms are the lumbar pain and the hematuria it presented. The pain is due to obstruction of the pyelo-ureteral junction and / or local extension. The hematuria may be due to the primitive tumor or to a lithiasis absent in our case [6]. The unseen infectious signs may be present and lead us away from the kidney cancer diagnosis, especially since the urine culture are often positive and point to a urinary infection [7]. The insidious progression of the disease and the absence of any signs or pathognomonic symptoms complicate the diagnosis delay as in other renal cancers [6]. The scanner is the reference imaging in the kidney tumors diagnosis; it has made it possible to suspect the diagnosis, to take the stock of the loco regional and remote extension.

The anatomopathological study made it possible to retain the diagnosis. Squamous cell carcinoma generally occurred as a single tumour, while the urothelial carcinomas showed the recognized tendency to multiplicity [8]. Histology showed malpighian epithelial differentiation of the entire tumor, unlike the diagnosis squamous inflexions frequently observed in urothelial carcinomas [9]. The component in-situ at the level of the pelvis epithelial lining was in favor of the tumor primitive nature [8] (Figure 4).

The urothelium chronic irritation of is presumed to be a cause of the malpighian metaplasia with subsequent malignant progression to cell carcinoma [2,5]. The common causes of chronic irritation in descending order are the long duration of a kidney stone development, chronic analgesic abuse, radiation therapy and schistosomiasis [6,7]. The prognosis of this type of malignant neoplasia remains bad with an average survival between 03 and 07 months and a survival 5-year one not exceeding 10% [5,6,10]. With this type of cancer, the prevention takes on all its importance. It is essential to treat kidney stones correctly and early, to prevent from recurrent upper urinary infections.

**Patient perspective**: during her hospitalization and after treatment, the patient was quite satisfied with the care she received and quite optimistic about the progress of her condition.

**Patient's consent**: informed consent was obtained from the client for us to use the pictures.

## Conclusion

Primary squamous cell carcinoma of the kidney is a rare tumor. The diagnosis is often fortuitous on a piece of nephrectomy. This neoplasia most often develops from squamous metaplasia of the urothelium and chronic irritation. Surgery offers the best healing option. It should be suspected in any patient who presents a renal tumor associated with any cause of squamous metaplasia of the urothelium even without a past history of renal lithiasis, recurrent urinary tract infection or urinary schistosomiasis.
